# Under COVID-19 Pandemic: A Quasi-Experimental Trial of Observation on Diabetes Patients' Health Behavior Affected by the Pandemic From a Coaching Intervention Program

**DOI:** 10.3389/fpubh.2021.580032

**Published:** 2021-05-14

**Authors:** Ching-Ling Lin, Li-Chi Huang, Yao-Tsung Chang, Ruey-Yu Chen, Shwu-Huey Yang

**Affiliations:** ^1^Department of Endocrinology and Metabolism, Cathay General Hospital, Taipei, Taiwan; ^2^Department of Internal Medicine, School of Medicine, College of Medicine, Taipei Medical University, Taipei, Taiwan; ^3^School of Public Health, Taipei Medical University, Taipei, Taiwan; ^4^School of Nutrition and Health Sciences, Taipei Medical University, Taipei, Taiwan; ^5^Nutrition Research Center, Taipei Medical University Hospital, Taipei, Taiwan; ^6^Research Center of Geriatric Nutrition, College of Nutrition, Taipei Medical University, Taipei, Taiwan

**Keywords:** COVID-19, pandemic (COVID-19), diabetes, health coaching, health behavior

## Abstract

**Introduction:** The aim of this study was to explore the impact of diabetes self-management and HbA1c affected by the COVID-19 pandemic and the epidemic prevention work.

**Methods:** This quasi-experimental study collected a pooled data from a randomized-control study between February and May 2020 in which 114 participants who presented type 2 diabetes were recruited. The intervention group had health coaching and usual care, whereas the control had usual care only. The main outcome variables of this observation study were the change of HbA1c, physical activity, and eating out behavior within this time interval.

**Results:** We found that the eating out behavior of both groups had decreased, and if a health coach helped the patients set physical activity goals in the two groups, the physical activity behavior will not be impacted due to the pandemic.

**Conclusions:** While every country is focusing on COVID-19 pandemic prevention, especially when strict home quarantine measures and social distancing are adopted, reminding and assisting chronic patients to maintain good self-management behavior may lessen the social and medical system burdens caused by the deterioration of chronic conditions due to the excessive risk prevention behavior and the epidemic prevention work.

**Trial Registration:** www.isrctn.com, identifier number: ISRCTN14167790, date: 12 July, 2019.

## Introduction

In December 2019, the new coronavirus disease (COVID-19) first broke out in China and spread around the world quickly in just a few months, killing tens of thousands of people (1, 2). Until now, even though millions of vaccine doses have been administered worldwide, there are still tens of thousands of new cases increasing every day, and many countries are still adopting varying degrees of pandemic prevention measures, such as strict restrictions on people going out and home quarantine (3). Many countries have adopted anti-pandemic measures to close schools and public entertainment venues, and they also encourage people to stay at home as much as possible to reduce the risk of disease transmission. The rapidly spreading and uncontrolled pandemic situation and strong anti-pandemic measures have not only caused huge pressure on medical staff, made people feel anxious and panicked, but also affected people's healthy living habits (4, 5). These changes also impact people with chronic diseases where their original lifestyles and self-management have disrupted.

Taiwan has made quite remarkable pandemic prevention results in this pandemic (6). Until March 2021, there were only about 1,007 confirmed cases and 10 deaths with no new confirmed local cases for over a month (1). As early as January 2020, Taiwan initiated stricter border control measures and put in place quarantine measures, as well as requiring people to wear masks when taking public transportation and control hospital access and visits. Although Taiwan did not implement a lockdown to combat the COVID-19 outbreak, during this period, it still experiences a significant drop in economic activity, with a number of companies allowing employees to work from home, many tourist attractions closed, and people feeling hesitated to go out. This may also affect chronic patients' willingness to have regular visits to the doctor and maintain healthy living habits. In fact, the number of outpatients and inpatients at all levels of hospital has declined significantly, about 14% lower than the same period last year (7).

In October 2019, we launched a diabetes health coaching program at Cathay General Hospital in Taipei, and it ended in August 2020. We tested the effectiveness of coaching on healthy lifestyle habits and mental health of diabetic patients in a randomized controlled trial. However, in the first few months of the COVID-19 pandemic, we found that the behavior of the participants seemed to be affected by the pandemic, such as avoiding going to hospital for regular screening and making regular visits, reducing outdoor physical activity, and changing eating habits, and in turn interfered with the validity of the research. Indeed, other countries also faced similar behavior changes when stricter lockdown measures were imposed (8–11). However, there are not many studies on impact of chronic patients during the pandemic, and most of them are still focused on related studies that chronic diseases increase the mortality and the risk of death from COVID-19 (12–14). Therefore, we intended to probe into the data to determine whether the pandemic would really affect the self-management behavior and health status of chronic patients, and to realize the possible impact of our health coaching program in the first few months of the pandemic. It may be one of the limitations of our original study program.

In this study, our aim was to observe the behavioral impact of the COVID-19 pandemic on diabetes patients and the interference with our original health coaching study. Data were collected for analysis between February and May 2020, when Taiwan was affected by the pandemic the most, and we especially focused on those indicators that might be affected by the pandemic situation and anti-pandemic measures.

## Materials and Methods

This quasi-experimental study originated from a 6-month long, single-blinded coaching intervention program. This two-armed, randomized-control trial was endorsed by the Institutional Review Board (IRB) of Cathay General Hospital. The two groups were: (1) coaching intervention every month on top of diabetes shared care or (2) diabetes shared care only. Data were collected at the baseline and at the end of 3- and 6-month intervention.

In this study, we gathered a pooled data between February and May 2020. It meant that the data comprised the 3- and 6-month follow-up patients. This paper was focused on the effect of risk perception behavior under COVID-19 pandemic; in other words, the data were not intended to validate the effectiveness of our intervention. In total, there were 18 patients in the intervention group and 13 in the control group, who had the 6-month follow-up. The period from February to May was chosen since the first confirmed COVID-19 diagnosis occurred in Taiwan on January 21, and various anti-pandemic measures were launched in February. During this period, every hospital had launched numerous anti-pandemic control measures in accordance with government policies, such as access control and limiting inpatient visits. In May, the pandemic prevention measures were gradually relaxed, and on May 10, it had reached a record of no new local cases for a month. Therefore, we believed that the period from February to May was when people in Taiwan began to feel anxious about the COVID-19 pandemic and gradually felt relieved.

### Study Procedure

#### Study Population and Recruitment

Participants were recruited from a medical center—Cathay General Hospital in Taipei, Taiwan, which is one of the highest levels of hospital accreditation in Taiwan. The first author screened potential patients with type 2 diabetes mellitus from the hospital database, randomly assigned them to the intervention group and the control group, and then two physicians who specialize in endocrine and metabolic disorders recruited them separately. To be considered for inclusion, only patients, between 20 and 75 years old, who were diagnosed with type 2 diabetes for at least 1 year and had an HbA1c of 7.0% or greater for the past 6 months, exhibiting no clinically significant depression or cognitive impairment were recruited. Patients in the intervention group were informed of the coaching program whereas the patients in the control group were informed of pre-posted survey. Upon gathering signed informed consent, the health coach then started interviews and data collection. Participant enrolment was carried out from October 2019 to February 2020.

#### Sample Size

A total of 47 participants in each group was required to establish a clinically meaningful difference in coaching intervention, defined as a 1% between-group difference in HbA1c and standard deviation of 1.7, with a probability of a type I error of 0.05 and a power of 80%. Factoring in a 20% dropout rate, we aimed at recruiting 60 in each study group.

#### Intervention

A detailed description of our health coaching intervention has previously been published (15). The coaching was provided by a single coach who had over 120 h certified coach training and received the International Coach Federation's (ICF) Associated Certified Coach (ACC) credential and a master's degree in public health. Patients in the intervention group had an initial face-to-face session, together with baseline measurement, and then was offered telephone coaching sessions monthly for 6 months. In the first session, the coach asked each participant to set his or her 6-month HbA1c goal and the first behavior change goal. The behavior goal must be one of the behaviors related to diabetes self-management, including physical activity, healthy diet, medical adherence, and/or regular self-monitoring blood glucose (SMBG). If a patient had more than one behavior change goal, the coach would ask him or her to prioritize the goals. The goal had to be designed to follow the “SMART” rule (i.e., specific, measurable, attainable, realistic, and timely). The coach would then record the goals set by the patient and the content of the coaching for follow-up and analysis. It made us to explore possible clues about patients' health behaviors affected by pandemic from the qualitative record.

Each patient in the control group would only receive a face-to-face coaching session and baseline measurement at the baseline without having any coaching call at all. Coaching also helped patients in the control group set a behavior change goal and encouraged them to carry it out. Both the intervention and control groups received diabetes health education and usual care based on the diabetes shared care network program of Cathay General Hospital. The diabetes health education was conducted by diabetes educators. All participants were allowed to contact diabetes educators to ensure acquiring adequate educational resources.

#### Outcome Measures

In this study, we collected variables which might be associated with or affected by COVID-19 pandemic and prevention policy. Hence, our outcome variables included HbA1c, physical activity, and eating out behavior.

HbA1c was measured using the patients' blood test when they had regular visits. Physical activity was assessed using the Godin leisure-time physical activity scale (16, 17). It marked the number of days in a week the patients did vigorous, medium and light physical activities. After weighing and summing up each level of physical activity, the higher the figure the more physical activities the patients did. The number of times eating out per week, which also included take-away food, was collected from the coaching record. Physical activity and health diet goal setting were also collected from the coaching record, and both of these two variables were binary variables.

The sociodemographic variables included gender, age, educational level, employment status, diabetes history, and basic SMBG habit (times per week).

#### Statistical Analysis

Chi-square tests or *t*-tests were used to assess differences in sociodemographic factors, health behaviors, and HbA1c between the two groups. Paired *t*-test was used to assess the difference in HbA1c, physical activity, and eating out behavior for each group, and *t*-test was also used to assess the difference of pre-post differences between the two groups. We used Mann-Whitney *U*-test and Wilcoxon signed-rank test to assess the sub-group differences in intervention and control group and the pre-post differences under each sub-group.

Fisher's exact test and Mann-Whitney *U*-test were used to assess difference between those patients dropped from regular visits in the intervention and control groups, and the difference between normal patients and dropped patients within the intervention and control group.

All tests were analyzed at a 95% significance level (*p* < 0.05). The analyses were conducted using PASW 20.0 software for Windows (SPSS, Chicago, IL).

## Results

### Baseline Data

**Figure 1:**
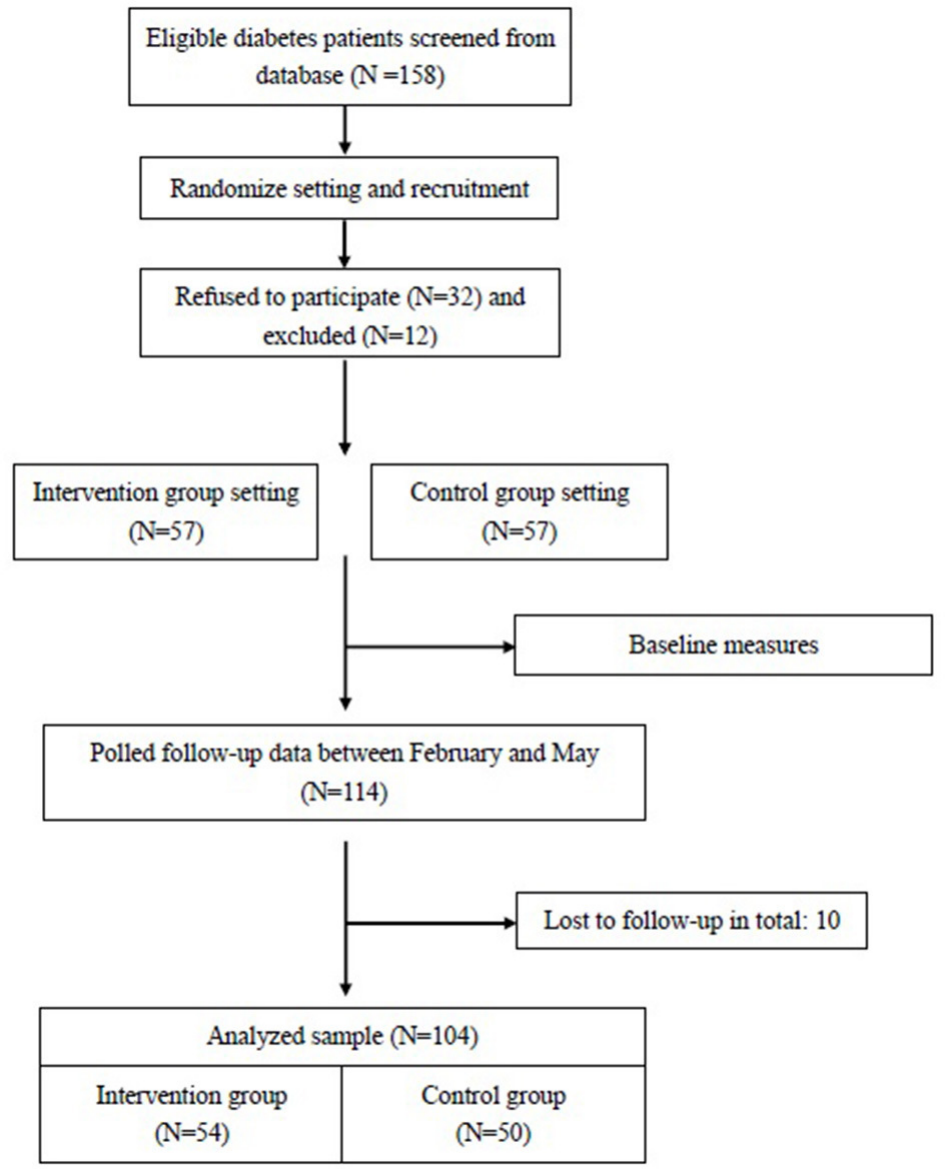
Flow diagram of participants: recruitment, intervention and follow-up.

Between October 2019 and February 2020, two physicians had invited 158 potential patients to participate and eventually a total 114 subjects were enrolled in the study randomly. Between February and May 2020, outcome measures were available for 54 patients (95% of the 57 patients) in the intervention group and for 50 patients (88% of the 57 patients) in the control group (Figure 1). In total, nine participants did not return regularly or ask family members to take prescription to avoid coming to the hospital, but no one withdrew from the study.

The demographic characteristics of the study groups are listed in Table 1. Of 114 participants, 48.2% were female, mean age was 62.2 years (SD = 8.73), 37.7% had a bachelor's degree or higher, 48.2% was retired, mean years of diagnosed diabetes was 13.5 years (SD = 7.66), mean HbA1c was 8.3% (SD = 1.08), and average 2.2 times of SMBG per week (SD = 2.29). There was no significant difference between these two groups in baseline characteristics, and there was also no significant difference between those patients who skipped their regular visits (gender, *p* = 0.667; age, *p* = 0.730; educational level, *p* = 0.217, employment status, *p* = 0.738, and diabetes history, *p* = 0.730).

**Table 1:** Demographic characteristics and baseline value of study groups.

	**Demographic characteristics** ***N*** **(%)**		***p*-value**
	**Intervention group (*n* = 57)**	**Control group (*n* = 57)**	
Gender			0.851
Male	30 (52.6)	29 (50.9)	
Female	27 (47.4)	28 (49.1)	
Age, years (mean ± SD)	61.28 ± 9.75	63.22 ± 7.50	0.242
Educational level			0.361
Junior high school or below	11 (19.3)	16 (28.1)	
Senior high school	20 (35.1)	24 (42.1)	
University	19 (33.3)	12 (21.1)	
Master's degree or above	7 (12.3)	5 (8.8)	
Employment status			0.574
Employed	31 (54.4)	28 (49.1)	
Retired	26 (45.6)	29 (50.9)	
Diabetes history (years, mean ± SD)	13.73 ± 7.73	13.16 ± 7.65	0.708
HbA1c (%, mean ± SD)	8.38 ± 1.29	8.14 ± 0.96	0.260
Physical activity points (mean ± SD)	13.42 ± 14.12	9.32 ± 12.80	0.139
SMBG (times per week, mean ± SD)	2.37 ± 2.40	2.04 ± 2.19	0.439

### Possible Effect of COVID-19 Pandemic

Overall, between February and May, the coaching intervention was associated with a significant decrease of 0.49% (CI = 0.24–0.75, *p* < 0.01), and a non-significant increase of 0.05% (CI = −1.12 to 0.29, *p* = 0.648) in HbA1c level was observed in the control group (Table 2). Both pre-test and post-test in HbA1c level between two groups were non-significant, but the difference of pre-post test was significant different between two groups (*p* = 0.002).

**Table 2:** Effect of COVID-19 pandemic to diabetes health coaching intervention program and health behaviors according to paired *t*-test and *t*-test.

	**Pandemic effect (mean ± SD)**		***p*-value**
	**Intervention group (*n* = 54)**	**Control group (*n* = 50)**	
**HbA1c, %**			
Pre-test	8.40 ± 1.29	8.19 ± 0.96	0.189
Post-test	7.99 ± 1.00^a^, ^b^	8.24 ± 1.10	0.248
**Physical activity points**			
Pre-test	13.19 ± 14.10	8.28 ± 9.82	0.048^*^
Post-test	15.22 ± 11.70	10.30 ± 10.28	0.113
**Frequency of eating out in a week, times**			
Pre-test	6.26 ± 5.66	5.75 ± 5.74	0.635
Post-test	4.49 ± 4.34^a^	4.10 ± 5.14^a^	0.686

* *p < 0.05*.

a *Significant difference between pre-post within the same group*.

b *Significant difference in difference between groups*.

Both the intervention and control groups were associated with a non-significant increase in physical activity, but the intervention group had a significantly more physical activity than the control group at baseline. Then we assessed the difference between different physical activity behavior change in the intervention group and the control group. Those patients with coaching intervention and chose to set physical activity goal had a significant increase in medium of nine points in physical activity indicator (IQR = 18.0, *p* = 0.007) (Table 3). On the contrary, patients in the intervention group without setting physical activity goal had a significant decrease (IQR = 3.0, *p* = 0.016). However, the pre-test data also showed those patients who did not make the choice of setting a physical activity goal had a significantly better physical activity habit in both the intervention group and the control group.

**Table 3:** The different between goal setting subgroups and coaching effects under COVID-19 pandemic.

	**Physical activity goal setting**					
	**Intervention group (*****n*** **= 54)**			**Control group (*****n*** **= 50)**		
	**Yes (*n* = 22)**	**No (*n* = 32)**	***p*-value**	**Yes (*n* = 19)**	**No (*n* = 31)**	***p*-value**
**Physical activity points**						
Pre-test [MD(IQR)]	0 (10.0)	21.0 (24.0)	<0.001^*^^a^	0 (10.0)	9.0 (18.0)	<0.001^*^
Post-test [MD(IQR)]	18.0 (14.0)	18.0 (24.0)	0.451	6.0 (15.0)	12.0 (21.0)	0.451
Pre-post difference [MD(IQR)]	9.0 (18.0)^*^^b^	0 (3.0)^*^	<0.001^*^	0 (7.0)^*^	0 (8.25)	0.712
	**Healthy diet goal setting**					
	**Yes (*****n*** **= 38)**	**No (*****n*** **= 16)**	***p*****-value**	**Yes (*****n*** **= 43)**	**No (*****n*** **= 7)**	***p*****-value**
**Frequency of eating out in a week, times**						
Pre-test [MD(IQR)]	5.0 (10.0)	5.5 (12.0)	0.915	6.0 (12.0)	0 (6.0)	0.094
Post-test [MD(IQR)]	4.0 (8.0)	5.5 (7.0)	0.335	5.0 (10.0)	0 (4.0)	0.070
Pre-post difference [MD(IQR)]	0 (2.25)^*^	0 (0.0)	0.242	0 (2.0)^*^	0 (1.0)	0.869

* *p < 0.05*.

a *Analyzed with Mann-Whitney U-test*.

b *Analyzed with Wilcoxon signed-rank test*.

In this study, health diet goal setting was about more balanced and healthy diets for the diabetes patients, rather than simply decreasing the number of times to eat out. Both the intervention and control groups had a significant decrease in the number of times of eating out in a week. However, the decrease in the frequency of eating out behavior was associated with the healthy diet goal setting while those without healthy diet goal have non-significant decrease in the number of times.

There were non-significant of the baseline characteristics between follow-up patients and those who skipped hospital visits during the intervention, and it was the same as in the control group.

## Discussion

In this study, we found that between February and May, the COVID-19 pandemic might cause the decrease in the patients' physical activity and eating out behavior. However, if any patient had set behavior change goal and even had follow-up communication, they were less affected by pandemic. Overall, the intervention group had the decrease in HbA1c by an average of 0.49% in these 3 months. Since the physical activity habit and healthy diet had considerable effect on blood control, helping patients maintain these good habits might be even more important during the COVID-19 pandemic.

In both the intervention and control groups, patients who originally had poorer physical activity habits were more likely to set change goals in coaching during pretest; however, patients with telephone coaching tracking, the effect of the change was significantly better. If the goal of physical activity habit change was not set, those who had better physical activity habits might reduce the amount of physical activity due to the pandemic. It was clear that they might need someone to help them make an appropriate decision to maintain good physical activity habit. Health coaching might have served an effective way to maintain or improve physical activity habit. Some studies have confirmed that health coaching can indeed help diabetes patients improve physical activity habits (18–20).

We also found that during the pandemic, the frequency of eating out was decreased among most of the subjects. Although the difference between the two groups was non-significant, patients who had set diet improvement goals had a significantly lower number of times eating out. Most diabetes health education, nutrition counseling, and health coaches provide guidance on dietary changes for diabetics mainly with dietary choices rather than eating out behavior, and these interventions also seemed to be indirectly affecting eating out habits (21, 22). Reducing the number of eating out means that patients have more chances to prepare their own food, but how to eat healthy is still the main issue.

Since early 2020, only a very small number of studies have specifically focused on the changes in living habits of people with type 2 diabetes during the pandemic, and the results of these studies seem to be inconsistent. A study in Spain found that people with type 2 diabetes have increased their intake of both vegetables and unhealthy snacks and decreased their physical activity during the lockdown (23). However, a study in India found that due to the lockdown policies, work pressure is reduced and it has helped people with type 2 diabetes improve their medication adherence and physical activity at home, thereby improving their blood sugar control (24). It seems that the changes in healthy lifestyle caused by the pandemic are varied in different countries (25), but many scholars still suggest that effective home physical activity and healthy eating strategies should be developed to help chronic patients maintain their health status (26, 27). During this period, telephone coaching or counseling can be a suitable and effective method as well (28, 29), and many studies have even confirmed the effectiveness of telephone coaching before the pandemic (30, 31). Therefore, our study results indeed verify that behavioral coaching should help people with type 2 diabetes maintain a good lifestyle during the pandemic and even continue to improve their blood sugar control.

Health coaching may effectively strengthen communication between doctors and patients, and at the same time strengthen sociopsychological support (32, 33). Of course, it may also help patients reduce unnecessary or excessive risk perception, strengthen their sense of responsibility of self-management, and encourage patients to regular visit the hospital for normal follow-ups to track diabetes and maintain appropriate healthy living habits according to the recommendations of the doctor (34, 35). Because of the lockdown and preventive measures, people's lifestyle and daily habits have changed. For example, instead of swimming and going to the gym people have switched to outdoor sports such as jogging and cycling (36); moreover, people often prepare their own food rather than eating out. However, before the pandemic, making changes in behavior and habits was already quite difficult, and now the imposed restrictions have made effective behavior coaching and guidance even more important. Hence, it is inadequate to rely on normal health education alone (37). Although the COVID-19 pandemic in Taiwan has gradually eased compared with other countries, making the result of this study less clear, we believe that in other countries where the pandemic is more serious, patients with chronic diseases who fail to maintain healthy lifestyle and regular follow-ups will create a huge burden to the society and medical system in the future. Therefore, given that the pandemic is expected to continue for some time, more rigorous interventional studies are still needed.

In this study, of those patients who dropped from regular visits, it seemed that there was no significant difference in patients' characteristics between the two groups. Although this might be because the sample observed in this study was not large enough, it could be interpreted that there were fewer patients in the hospital and more people chose not to visit the hospital during the pandemic. This is consistent with the findings of some studies (11, 38), which means that during the epidemic, it is still important to assist chronic patients to maintain regular visits.

This study has several strengths. First, this is one of the few studies done on health behavior changes in patients with chronic diseases in Taiwan during the pandemic. Although this study is not specifically designed to explore the behavior changes of Taiwanese people under the pandemic and has some limitations due to our statistic method, it can still reflect to a certain degree the effect of the pandemic at that time. Second, this article is specifically for the observation in the early months of the pandemic. Few studies were able to promptly explore the changes in people's living habits at the beginning of the chaos. Therefore, even if the interpretation of the research results is relatively limited, it can still provide valuable observations and discoveries.

Of course, this study has some limitations. First, since we pooled data with two different follow-up periods, it was not possible to accurately compare the pure effect of coaching on the maintenance of diabetes self-management on patients. Hence, detecting the pure difference of HbA1c was also unsuitable. Second, since this study was orientated from a behavior intervention study which focused on patients with type 2 diabetes rather than an observational study; therefore, it had fewer study samples than observational studies and did not compare with a healthy control group. It made the evidence less strong from this quasi-experimental study and had lower reproducibility than normal well-designed observational studies, but we believe it still has considerable reference value when a future outbreak occurs, especially in the early stage of the pandemic, since it can happen too suddenly for people to have a well-designed and rigorous study specifically focusing on the change of the lifestyle of chronic patients under the pandemic, especially at the initial stage.

According to the result of this study, here are some applications. First, during the pandemic prevention period, policy makers should also pay attention to the communication need of chronic patients to lessen excessive risk avoidance by actively offering counseling and intervention programs instead of passively handing out patient guides or providing health education. The pandemic has drastically changed people's lifestyles, so how to assist patients in adjusting and maintaining healthy living habits during the pandemic is still an important issue. People's lifestyles have changed considerably after the outbreak; therefore, assisting people in implementing a healthy lifestyle can reduce the impact of lifestyle changes. For example, we can especially promote some physical activities that are suitable for the home environment, introduce healthy cooking ways, encourage chronic patients for regular screening and visits, or use telemedicine to handle the current status of patients. Second, good communication and establishing a good relationship with patients can encourage patients to use the correct channels to obtain pandemic-related information more effectively and resolve patients' queries; hence, it can avoid risky behaviors, especially skipping regular hospital visits. We believe that if the COVID-19 pandemic does continue to spread for some time, these works may avoid the accidental deterioration of chronic disease control due to pandemic situation and pandemic prevention and will be very pivotal for medical system, government of all countries and the world.

## Data Availability Statement

The raw data supporting the conclusions of this article will be made available by the authors, without undue reservation.

## Ethics Statement

The studies involving human participants were reviewed and approved by Institutional Review Board (IRB) of Cathay General Hospital. The patients/participants provided their written informed consent to participate in this study.

## Author Contributions

S-HY, C-LL, R-YC, L-CH, and Y-TC participated in the conception, design of the study, performed the statistical analysis, and drafted the manuscript. C-LL screened eligible patients, then C-LL and L-CH recruited patients. Y-TC participated in acquisition of data. All authors contributed to the article and approved the submitted version.

## Conflict of Interest

The authors declare that the research was conducted in the absence of any commercial or financial relationships that could be construed as a potential conflict of interest.

## Abbreviations

COVID-19: Corona virus disease-2019
ICF: International Coaching Federation
ACC: Associate Certified Coach
HbA1c: Hemoglobin A1c
SMBG: Self-monitoring blood glucose
IRB: Institutional Review Board
SD: Standard deviation
IQR: Inter-Quartile Range
CI: Confidence Interval.
